# Diabetic macular morphology changes may occur in the early stage of diabetes

**DOI:** 10.1186/s12886-016-0186-4

**Published:** 2016-01-18

**Authors:** Yanwei Chen, Jianfang Li, Yan Yan, Xi Shen

**Affiliations:** The Department of Ophthalmology, RuiJin Hospital Affiliated Shanghai Jiao Tong University School of Medicine, Shanghai, China

**Keywords:** Diabetes retinopathy, Optical coherence tomography, Retinal morphology

## Abstract

**Background:**

The purpose of this study was to observe whether invisible morphological changes are presented in the two types of diabetes mellitus patients without diabetic retinopathy.

**Methods:**

Twenty-six type 1 diabetes mellitus (T1DM) patients and 34 type 2 diabetes mellitus (T2DM) patients without diabetic retinopathy (DR) were recruited for this study. They underwent complete examinations that included stereoscopic color fundus photography and optical coherence tomography (OCT). The OCT patterns were used to measure the macular retinal thickness (RT), the ganglion cell and inner plexiform layer (GC-IPL) complex thickness, the inner nuclear layer (INL) thickness, the outer nuclear layer (ONL) thickness and the subfoveal choroidal thickness (SFCT) using the enhanced depth imaging (EDI) patterns and the retinal fiber layer (RNFL) thickness around the optic disc. All results were compared to those of age- and sex-matched control groups.

**Results:**

In the patients with T1DM, the mean RT and GC-IPL complex thicknesses were significantly thinner than those of the control group (*p* < 0.05). The RNFL was found to be thinner at the 9 o’clock position around the optic disc in the patients compared with the control group. The SFCTs were similar in the controls and subjects. The INL and ONL were decreased in parts of the pericentral and peripheral areas in the T1DM patients (*p* < 0.05) and increased in the T2DM patients (*p* < 0.05).

**Conclusions:**

This study demonstrated that in short-duration T1DM patients, the layers of the retina are affected and that the neural tissue has begun to be lost. As diabetes develops, neurodegeneration may cause vascular permeability, which causes thickening of the retinal layers.

## Background

The global incidence of diabetes mellitus (DM) is increasing, and the number of patients is expected to reach 3.66 billion by 2030 [1]. Diabetic retinopathy (DR) is one of the most common and severe complications of DM and is also the leading cause of blindness. In America, 40 % of type 2 diabetes mellitus (T2DM) patients and 86 % of type 1 diabetes mellitus (T1DM) patients suffer from DR [2, 3]. In China, the incidence of DR is 27.9 % in urban areas and 43 % in rural areas [4, 5]. Thus, the early detection of DR is important for the preservation of useful acuity in later life.

The present examinations of DR included direct ophthalmoscopy, fundus photography, optical coherence tomography (OCT) and fluorescence angiography. OCT is a noninvasive technique that was developed to be rapid in terms of its hardware and software attributes, and OCT exhibits high levels of accuracy and definition in the detection of early and small changes in retinal morphology. The newly updated Carl Zeiss OCT software version 6.0 integrates FastTrac retinal tracking, FoveaFinder, ganglion cell analysis, ONH and RNFL OU analyses, enhanced depth imaging (EDI) and other powerful functions that enable the acquisition of reliable data for clinical research.

In addition to the retinal layers, the choroid has also attracted much interest due to its vascular structure and supply of oxygen and nutrients to the outer retina. The EDI pattern is also useful for clearly displaying choroid thickness and structural abnormalities [6]. Increases and decreases in macular and choroid thicknesses have been observed in patients with and without diabetic retinopathy in previous studies, and different mechanisms have been found to be responsible for these changes [7–11]. Our study aimed to fully utilize OCT to identify the types of morphological changes in DM patients without retinopathy.

## Methods

### Participants

This was a cross-sectional, case–control study. The design of this study followed of the principles of the Declaration of Helsinki and was approved by the Institutional Review Board of the RuiJin Hospital. Written informed consent was obtained from all participants or their parents (children who were under 16 years old). The patients were recruited from the outpatient clinic of the department of Endocrinology Medicine of the RuiJin Hospital affiliated with the Shanghai Jiao Tong University School of Medicine (Shanghai, China) between October 2013 and November 2014. The patients were diagnosed with type 1 or type 2 diabetes mellitus by an endocrine specialist and were treated with insulin or other medicines. The normal control subjects were selected from the medical examination center of the same hospital. They were age- and sex-matched to the patients; were free of all diagnoses of ocular disease, diabetes, or other systemic disease; and underwent a complete ophthalmologic examination that included a visual acuity assessment, slit-lamp examination with a handheld lens (Super Field; Volk Optical, Inc., Mentor, OH), intraocular pressure (IOP) assessment with a noncontact tonometer, and refraction, color fundus photograph (TRC-50IX; Topcon Corp.), and Cirrus high-definition optical coherence tomography (HD-OCT, Carl Zeiss Meditec, Dublin, California). The inclusion criteria were as follows: 1) a diagnosis of T1DM or T2DM with no clinical retinopathy as observed through dilated pupils by an experienced doctor using a stereoscopic slit lamp with a handheld lens; 2) a best-corrected visual acuity ≥20/25; and 3) refractive errors below -6 or above +3 diopter equivalent spheres. The exclusion criteria were significant media opacity, glaucoma, uveitis, ocular trauma or surgery history, and other serious systemic diseases. Additionally, age, sex, duration of diabetes (in years) were recorded, and fasting glucose and HbA1c levels were collected from the patients’ recent blood reports (Tables 1 and 2).

**Table 1:** Characteristics of the subjects

Parameters	T1DM	Control	*p* value	T2DM	Control	*p* value
Female/Male	17/9	14/11	0.572	18/16	23/13	0.467
BCVA	0.00(log MAR)	0.00(log MAR)	0.922	0.05(log MAR)	0.05(log MAR)	0.669
IOP, mmHg	17.53±4.27	16.27±3.03	0.357	14.54±2.22	16.07±3.41	0.834
Age, years	21.8±5.3	23.1±4.8	0.397	59.7±13.9	57.0±7.5	0.183

All values are presented as the mean ± SD, and differences were considered statistically meaningful when *p* < 0.05

**Table 2:** Differences between T1DM and T2DM patients

Parameters	T1DM	T2DM	*p* value
Fasting blood glucose, mg/dL	6.22±1.30	6.37±1.37	0.248
HbAlc, %	7.84±2.39	6.56±0.65	0.072
DM duration, years	2.1±3.0	10.17±6.84	0.000*

All values are presented as the mean ± SD

*Statistically meaningful

### OCT measurements

**Fig. 1:**
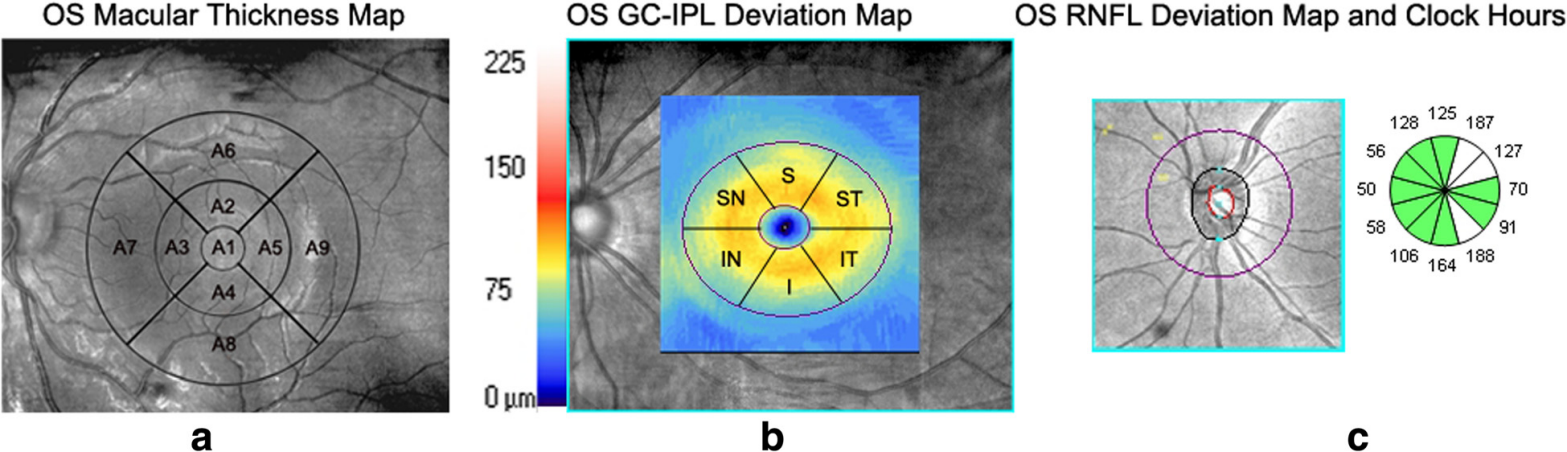
**a**. Macular retinal thickness map of nine zones of the left eyes based on OCT. **b**. Ganglion cell and inner plexiform layer complex thickness maps of nine zones of the left eyes based on OCT. **c**. Retinal nerve fiber layer deviation map and clock positions around the optic discs of the left eyes based on OCT

A Cirrus HD-OCT 4000 (software Version 6.0) was used to obtain the macular images. The patients placed their chins in a chinrest with their foreheads contacting a headrest and fixated on a green target. The macular cube 512 × 128 scan protocol was used to acquire all 128 OCT B-scans that covered an area of 6 × 6 mm and were 2 mm in length centered on the fovea. A mean retinal thickness (RT) map of nine zones from the internal limiting membrane to the retinal pigment epithelium was automatically measured. We defined the foveal area (A1) as a central circle with a diameter of 1 mm, the pericentral area (A2-A5) as a donut-shaped ring centered on the fovea with an inner diameter of 1 mm and an outer diameter of 3 mm, and the peripheral area (A6-A9) as a ring with an inner diameter of 3 mm and an outer diameter of 6 mm (Fig. 1a).

The ganglion cell analysis algorithm was used to measure the GC-IPL thickness within a 14.13 mm^2^ elliptical annulus centered on the fovea based on the 3-dimensional data generated from the macular cube 512 × 128 scan protocol. The outer boundary of the retinal nerve fiber layer (RNFL) and the outer boundary of the inner plexiform layer were automatically segmented by the algorithm. The GC-IPL thicknesses of the superotemporal, superior, superonasal, inferotemporal, inferior and inferonasal sectors were automatically measured (Fig. 1b).

**Fig. 2:**
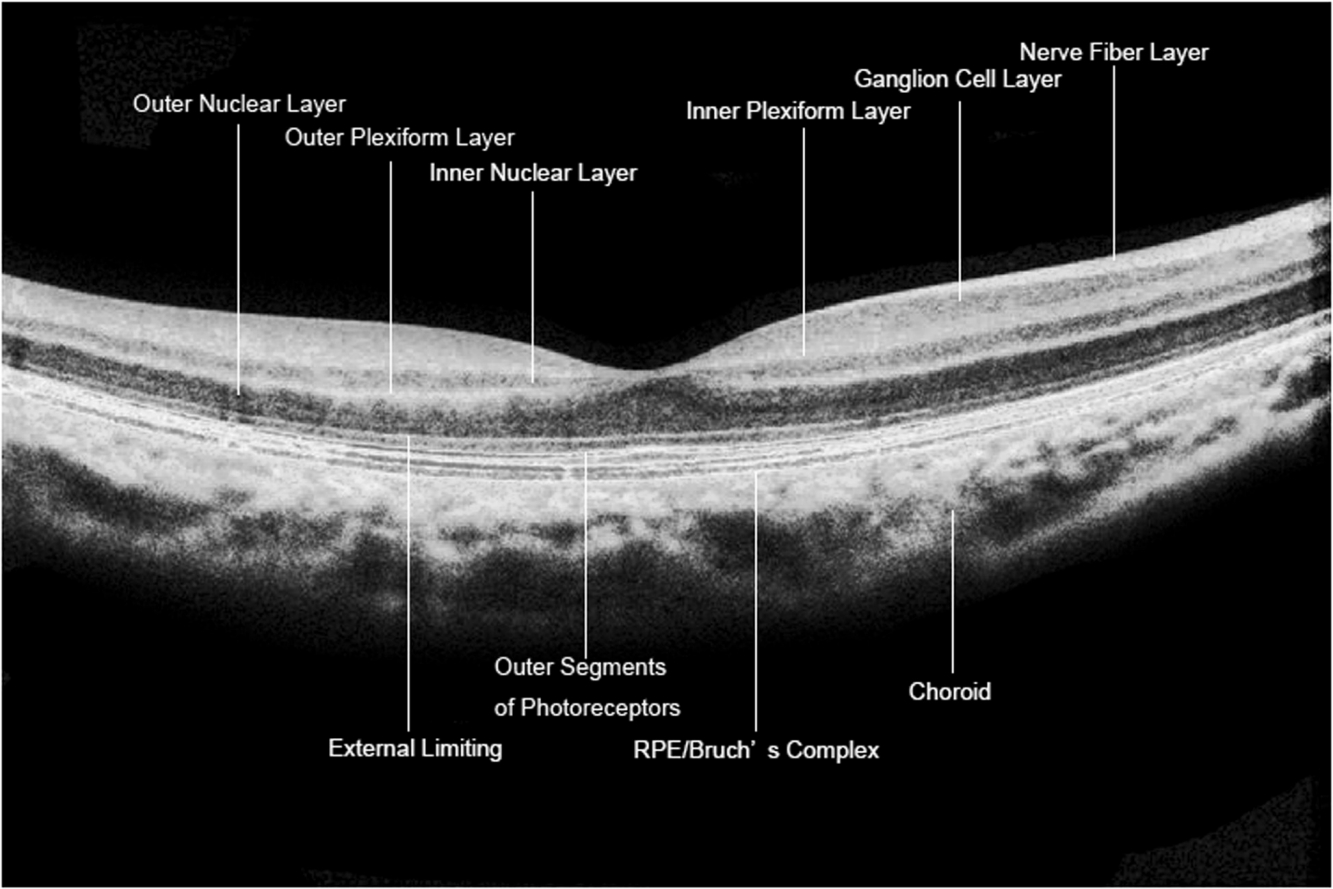
Retinal layers shown in an OCT EDI pattern scan image

A one-line raster scan of the EDI pattern was performed to obtain high-quality images of the retina and macular choroid. The scan line was as close as possible to the fovea in the horizontal (0°) and vertical (90°) directions. Only images with signal strengths exceeding 6 were selected for the study. The thicknesses of the inner nuclear layer (INL) and the outer nuclear layer (ONL) were measured manually every 0.5 mm from the center to the periphery in the nasal and temporal directions. Each measurement was performed three times, and the mean values were determined. The choroid thickness was defined as the vertical distance from the hyper-reflective line of Bruch’s membrane to the hyper-reflective line of the inner surface of the sclera. The subfoveal choroidal thickness (SFCT) was measured in the horizontally and vertically scanned images to obtain a mean value (Fig. 2).

The optic disc cube scan protocol was used to obtain the RNFL measurements from the T1DM patients. There were 200 A-scans, and from each of 200 B-scans, the software automatically determined the center of a disc with a radius of 1.73 mm. The RNFL thicknesses of 12 sectors of the optic disc that were defined like the hours on a clock were measured and calculated using this software. The data were recorded in the superior-to-temporal, inferior-to-nasal, and clockwise directions (Fig. 1c).

### Statistical analysis

Statistical analyses were performed using SPSS software version 20.0 for IOS (IBM Corporation, Chicago, IL, USA). All data are expressed as the mean ± standard deviation (SD). A chi square test was used to assess the difference in the female/male ratio between the patients and controls. Analysis of variance (ANOVA) was used to assess the differences in mean age, BCVA, IOP, fasting glucose level, HbA1c and DM duration between the patients and controls. The mean RT, GC-IPL complex, INL, ONL and RNFL thicknesses in the diabetic patients and controls were compared using independent *t* tests. The results were considered statistically significant when *p* < 0.05.

## Results

**Fig. 3:**
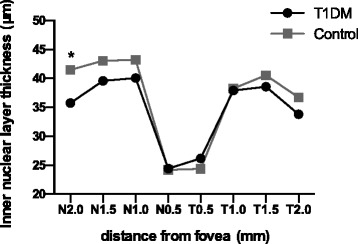
Comparison of the inner nuclear layer thicknesses in the perifoveal area between the type 1 diabetes patients and the control group. *Statistically meaningful

**Fig. 4:**
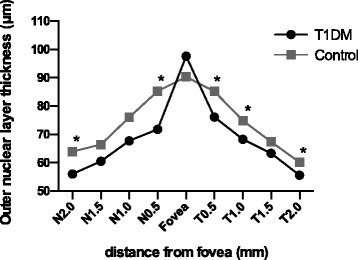
Comparison of the outer nuclear layer thicknesses in the perifoveal area between the type 1 diabetes patients and controls. *Statistically meaningful

**Fig. 5:**
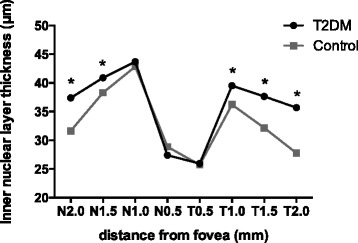
Comparison of the inner nuclear layer thicknesses in the perifoveal area between the type 2 diabetes patients and controls. *Statistically meaningful

**Fig. 6:**
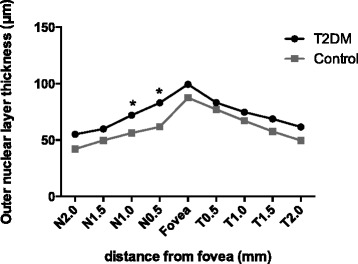
Comparison of the outer nuclear layer thicknesses in the perifoveal area between the type 2 diabetes patients and controls. *Statistically meaningful

In total, 26 T1DM patients and 34 T2DM patients were analyzed in this study. Age- and sex-matched controls were 25 and 36 respectively (Table 1). Both eyes of all participants were scanned, and the left eyes were selected for analysis. The mean RTs of all nine of the zones of the T1DM subjects were thinner than those of the control group, and the differences in zones A2 to A9 were statistically meaningful (*p* < 0.05); however, no significant changes were found in the T2DM patients (Table 3). All six sectors of the GCIPL complex in the T1DM patients were thinner than those in the control group, but these values were similar in the T2DM patients and controls (Table 4). The INL and ONL of the T1DM patients were both thinner at some of the tested points in the periphery and pericentral areas (*p* < 0.05) and slightly thicker in the fovea; the latter difference was not statistically meaningful (Figs. 3 and 4). In contrast, the opposite pattern of results was observed in the T2DM patients who displayed thicker layers (Figs. 5 and 6).

**Table 3:** The retinal thicknesses in nine zones in type 1 and type 2 DM patients and controls

	Type 1 DM	Control	*p* value	Type 2 DM	Control	*p* value
Superior	80.72±9.65	85.50±6.05	0.044*	82.74±8.91	85.24±6.47	0.316
Superotemporal	78.64±8.78	85.17±7.45	0.007*	81.13±8.19	82.59±6.29	0.527
Inferotemporal	80.00±7.09	85.29±5.29	0.005*	81.81±8.29	82.47±6.41	0.776
Inferior	78.28±7.01	82.35±6.34	0.041*	79.13±7.56	81.12±5.94	0.354
Inferonasal	79.96±6.49	84.63±6.61	0.016*	80.03±11.58	82.94±5.99	0.340
Superonasal	82.96±5.95	86.71±6.98	0.049*	84.19±10.49	86.59±6.54	0.399

All values are presented as the mean ± SD

*Statistically meaningful

**Table 4:** Thicknesses of the ganglion cell layer and inner plexiform layer in six sectors in the type 1 and type 2 DM patients and controls

	Type 1 DM	Control	*p* value	Type 2 DM	Control	*p* value
Superior	80.72±9.65	85.50±6.05	0.044*	82.74±8.91	85.24±6.47	0.316
Superotemporal	78.64±8.78	85.17±7.45	0.007*	81.13±8.19	82.59±6.29	0.527
Inferotemporal	80.00±7.09	85.29±5.29	0.005*	81.81±8.29	82.47±6.41	0.776
Inferior	78.28±7.01	82.35±6.34	0.041*	79.13±7.56	81.12±5.94	0.354
Inferonasal	79.96±6.49	84.63±6.61	0.016*	80.03±11.58	82.94±5.99	0.340
Superonasal	82.96±5.95	86.71±6.98	0.049*	84.19±10.49	86.59±6.54	0.399

All values are presented as the mean ± SD

*Statistically meaningful

The SFCTs were 294.44 ± 60.96 μm in the T1DM patients and 303.16 ± 60.23 μm in the T1DM control group (*p* = 0.64). In the T2DM patients and their control group, these values were 284 ± 84.89 μm and 254 ± 67.36 μm, respectively (*p* = 0.32).

The RNFL thicknesses of the optic discs of the T1DM patients were significantly decreased at the 9 o’clock position (*p* = 0.005).

## Discussion

Previous studies have reported changes in the macular retinal thickness and the thicknesses of different layers in patients with and without diabetic retinopathy [7–11]. The early diagnosis and early detection of functional changes related to DR that occur prior to retinal morphology changes are important for preventing DR. Some researchers have found that retinal neurodegeneration may occur in DR before any microcirculatory abnormalities can be detected [12, 13]. Neural apoptosis and the loss of ganglion cell bodies and glial reactivity are now considered to be the main factors in DR [14, 15].

In this study, we observed significant decreases in the RTs of the A2 to A9 zones and the thickness of all sectors of the GC-IPL complex in the macular zones of the T1DM patients. Additionally, among the tested points, N2.0 in the INL and N0.5, N2.0, T0.5, T1.0, and T2.0 in the ONL were found to be decreased in the patients. In the RNFL, the 9 o’clock position, i.e., the nasal section, was found to be reduced in the patients. In sharp contrast to the T1DM group, we did not observe many positive findings in the T2DM patients. No measured RT or GC-IPL complex thicknesses were found to be different between the T2DM patients and controls. However, increased thicknesses in the testing points N1.5, N2.0, T1.0, T1.5, and T2.0 in the INL and N0.5 and N1.0 in the ONL were observed. In the SFCT, we did not observe any changes in either the T1DM or T2DM patients.

To our knowledge, there were no previous ophthalmological studies of newly diagnosed T1DM patients. In the present study, the mean disease durations were 2.1 ± 3.0 years for the type 1 DM patients and 10.17 ± 6.84 years for the type 2 DM patients (*p* < 0.05). The T2DM patients exhibited no significant differences in the measures of retinal thickness or the GC-ILP complex, but these measures were decreased in the T1DM patients. Similar results have been reported in other studies [8, 9], which have detected either thickening or thinning of the retina. The majority of these reports found that the retina becomes thinner when DR is present [9, 16]. Together, all of the above-mentioned findings lead to the conclusion that the retinal and ganglion cell layer thicknesses are correlated with DM duration. In the initial few years of diabetes, the macular thickness decreases due to neural tissue loss, but as diabetes progresses, these thicknesses gradually increase due to vascular permeability in the retina [10, 11]. In patients without DR, Bronson-Castain et al. [17] found that the RT was decreased in a group of adolescent T2DM patients with a mean disease duration of 2.1 years. However, the authors of this study expressed doubt about the neural tissue theory based on the short duration of DM in their adolescent patients.

Our results indicated that the neurodegenerative effects on the retina probably occurred prior to vasculopathy in the early stages of diabetes based on the variations in the ages and disease durations of our population. Therefore, our results do not mean that there were no changes in the RTs of the T2DM patients; rather, the thickening of other layers, such as the INL and ONL, which were observed in the present study, probably masked these changes.

Due to technical limitations, we were unable to directly measure the ganglion cell layer thickness, but the newly updated OCT software provides accurate data about the GC-IPL complex for clinical studies.

Other studies have also observed ganglion cell loss in DR and nonproliferative diabetic retinopathy (NDR) patients [10], and axonal loss may lead to the thinning of the RNFL [18]. Moreover, both ganglion cells loss and axonal loss are correlated with HbAc1 and duration [19] and worsen as disease severity increases. The pericentral macular area has been found to be significantly thinner in patients with minimal DR [18, 20] and thicker in patients with preproliferative diabetic retinopathy [19], which suggests that neuronal abnormalities may precede vascular abnormalities [11].

We found that the RNFL around the optic disc was thinner in the patients than in the controls only at the 9 o’clock position, which may prove that RNFL degeneration occurs in the onset stage of diabetes [14, 21]; however, it is difficult to determine which area is the first to be affected.

In the inner nuclear layer, the pericentral and peripheral macular thicknesses were decreased in the T1DM patients compared to the controls, whereas they were thicker in the T2DM group, and the same pattern of alterations was observed in the outer nuclear layer. These results confirm our speculation that neurodegeneration occurs during the early stages of disease and that the vascular permeability increases with disease duration and leads to increased retinal thickness. However, the differences were not significantly meaningful at all of the tested points; thus, we may need to increase our sample size. Only manual measurements and horizontal EDI scans were used to determine the INL and ONL thicknesses, which limited our statistical power. Continuous follow-up of these subjects is also needed for further study.

The reports regarding choroidal thickness (CT) in diabetes patients seem to be consistent in that they have all reported decreased CTs in subjects with proliferative diabetic retinopathy or diabetic macular edema and a lack of significant changes in NDR patients [22, 23] and T1DM children without DR [24]. The breakdown of the blood-retinal barrier, loss of retinal vasculature integrity and the presence of hemodynamic abnormalities may contribute to changes in the choroidal thicknesses of diabetic eyes [25, 26]. In our study, the SFCTs of the T1DM and T2DM patients were similar to those of the controls, which may suggest that the choroid tissue is affected later than the neuroretinal layer in diabetic retinopathy.

### Conclusion

We detected morphological changes in DM patients with Cirrus HD-OCT, and the remarkable results observed in the T1DM patients confirmed that the loss of neural tissue begins in the early stages of diabetes. As diabetes develops, neurodegeneration may be masked by changes in vascular permeability that cause thickening of the retinal layers.

## Abbreviations

BCVA: best-corrected visual acuity
DR: diabetic retinopathy
EDI: enhanced depth imaging
GC-IPL: ganglion cell and inner plexiform layer
INL: inner nuclear layer
IOP: intraocular pressure
NDR: nonproliferative diabetic retinopathy
OCT: optic coherence tomography
ONL: outer nuclear layer
RNFL: retinal fiber layer
RT: retinal thickness
SFCT: subfoveal choroid thickness
T1DM: type 1 diabetes mellitus
T2DM: type 2 diabetes mellitus
